# Mycophenolate mofetil versus azathioprine as a first-line treatment for autoimmune hepatitis: a comparative systematic review and meta-analysis

**DOI:** 10.1186/s12876-025-04206-1

**Published:** 2025-08-22

**Authors:** Amani M. Ali, Mohamed E. Abdelrahim, Aya M. AbdelMagid

**Affiliations:** 1 Clinical Pharmacy Department, Faculty of Pharmacy, Cairo University, P.O. Box: 11562, Giza, Egypt; 2 Clinical Pharmacy Department, Faculty of Pharmacy, Beni-Suef University, Beni-Suef, Egypt

**Keywords:** Mycophenolate mofetil, Azathioprine, Autoimmune, Hepatitis

## Abstract

**Background:**

Autoimmune hepatitis (AIH) is a chronic progressive inflammatory liver disease of immune-mediated origin, which causes long-term liver inflammation and damage. Traditionally, treatment includes azathioprine (AZA) combined with steroids, but recent studies have highlighted mycophenolate mofetil (MMF) as a potential alternative, particularly for patients who do not respond well to AZA.

**Aims:**

This study aimed to evaluate the efficacy and safety of MMF versus AZA combined with steroids as first-line treatment for the management of AIH patients.

**Methods:**

A comprehensive systematic review was conducted using the keywords: mycophenolate mofetil, Cellcept, and autoimmune hepatitis. Studies comparing MMF to AZA combined with steroids in treatment-naïve patients with AIH were included. Efficacy was assessed based on complete biochemical remission (CBR), non-response, and relapse rates. Safety was evaluated on the basis of the incidence of serious adverse effects that led to treatment discontinuation. Statistical analysis included calculation of odds ratios (OR) and 95% confidence intervals (CI).

**Results:**

Out of 344 search results, four studies met the inclusion criteria, encompassing a total of 512 patients. MMF combined with prednisolone significantly improved both short-term CBR rates (OR, 2.56; 95% CI, 1.18–5.55) and long-term CBR rates (OR, 5.51; 95% CI, 1.7–17.91) compared to AZA combined with prednisolone. Furthermore, MMF treatment was associated with a significantly lower occurrence of serious adverse events (OR, 0.15; 95% CI, 0.07–0.34).

**Conclusions:**

Compared to AZA-based regimens, MMF-based first-line therapy for AIH appears to be a more promising, effective, and safe treatment option, yielding higher CBR rates and fewer serious adverse events requiring treatment discontinuation.

**Supplementary Information:**

The online version contains supplementary material available at 10.1186/s12876-025-04206-1.

## Introduction

Autoimmune hepatitis (AIH) is a rare, chronic, progressive, immune-mediated disease that affects the liver, leading to prolonged inflammation and liver damage, and is characterized by considerable heterogeneity [1–3]. This disease affects individuals of any age or ethnic background, with a higher prevalence among adult females and girls [4, 5]. However, advanced AIH is more commonly observed in male patients at the first evaluation and is associated with poorer outcomes than in females [6]. Recently, AIH has been reported with highest prevalence in countries with high human development indices, among elderly patients, at high latitudes of more than 45 degrees, and within North American populations [4, 7, 8]. The diagnosis of AIH is characterized by elevated levels of serum immunoglobulins, aspartate aminotransferase (AST), alanine aminotransferase (ALT), and one or more characteristic circulating autoantibodies, the histological presence of portal or lobular hepatitis with absence of viral hepatitis markers, and the responsiveness to immunosuppressive therapy [4–6, 9, 10]. However, owing to the variability in transaminase levels, accurate diagnosis is confirmed through liver biopsy and the detection of autoantibodies [6]. The clinical manifestations of symptomatic AIH typically include chronic liver disease symptoms, fatigue, abdominal discomfort, aching joints, itching, jaundice, enlarged liver, nausea, and spider angiomas on the skin [2, 11]. Additionally, patients may be asymptomatic or present with a fulminant form of the disease, such as acute severe hepatitis or acute hepatic failure [9]. If untreated, AIH can progress to complications such as ascites, fibrosis, cirrhosis, hepatic failure, and death [9, 11, 12]. Therefore, early diagnosis is important to prevent a worse prognosis [3, 6].

Despite the unknown etiology, it is believed that the disease may develop due to certain genetic factors that, when present, interact with unknown environmental factors to trigger a T cell-mediated immune reaction that attacks hepatocytes [11, 12]. Therefore, immunosuppression and suppression of inflammatory activity are crucial in AIH treatment and should be started as soon as the diagnosis is confirmed to be lifesaving and to prevent disease progression [5, 13]. The duration of immunosuppressive therapy should be at least 3–5 years, and continued for at least 2 years after the achievement of complete biochemical remission (CBR) with confirmatory liver biopsy results before treatment discontinuation [6]. The combination of high doses of prednisone or prednisolone and azathioprine (AZA) to induce and maintain remission is considered the first-line and standard of care (SOC) treatment for AIH management [13, 14]. However, major and serious concerns have evolved regarding such recommendations, indicating a therapeutic bias [6].

In 2020, the guidelines of the American Association for the Study of Liver Diseases recommended second-line treatment regimens for patients who experienced failure or intolerance to SOC treatment. Among these, mycophenolate mofetil (MMF) is a promising salvage therapy for AZA when combined with prednisolone [9]. Zachou et al. [15] reported a persistent 75% remission rate in patients with AIH after complete withdrawal of MMF/prednisolone following their treatment for a median period of 24 months. In 2019, a meta-analysis focusing on first-line therapies evaluated the efficacy of MMF against SOC for AIH in eight studies, which included not only treatment-naïve patients, but also those unresponsive or intolerant to SOC treatment. The MMF/prednisone combination proved superior to SOC (prednisone/AZA) in normalizing the serum levels of ALT, AST, and immunoglobulins and reducing treatment failure rates [5]. In this review, we aimed to assess the efficacy and safety of MMF versus AZA, combined with steroids, as first-line treatment for the management of AIH in treatment-naïve patients.

## Methods

This study followed the Preferred Reporting Items for Systematic Reviews and Meta-Analysis (PRISMA) statement guidelines [16]. A comprehensive search for evidence was conducted across multiple databases, including MEDLINE via PubMed, Cochrane Central Library, and Google Scholar. Furthermore, trial registries such as clinicaltrials.gov and the World Health Organization International Clinical Trials Registry Platform (WHO-ICTRP) were searched, with no restrictions on the study language or publication date. The keywords used included autoimmune hepatitis, mycophenolate mofetil and Cellcept [17]. The initial search was conducted in June 2024, and the last search was performed on July 2nd, 2024. A detailed search strategy is presented in Supplementary Table 1.

Studies were imported into Mendeley Reference Manager version 2.120.3. After duplicate removal, the titles and abstracts were screened by two independent reviewers (A. A. and A. A.) for further full-text consideration according to the inclusion and exclusion criteria. Discrepancies were resolved through discussion or consultation with a third reviewer (M. A.). The inclusion in the current review considers full-text articles comparing MMF/prednisone (or prednisolone) to SOC treatment (AZA/prednisone (or prednisolone)) with at least one outcome of interest. Patients treated with monotherapies such as prednisolone, AZA, or MMF were excluded from the quantitative analysis. The primary outcome was the efficacy of MMF as a first-line treatment for achieving CBR, defined as the ability to normalize immunoglobulin G and transaminase levels [3]. CBR was considered short-term if assessed within 6 months after starting the treatment and long-term if assessed after one year or more. Other outcomes included non-response rates, relapse rates and medication safety, with non-response defined as a < 50% decrease in aminotransferase levels at 4 weeks post-treatment initiation. Relapse was defined as any significant increase in aminotransferases and/or immunoglobulins at any time point after prior CBR [3]. Safety was assessed on the basis of the rate of adverse events that required treatment discontinuation. Only studies with separate results in treatment-naïve patients were considered.

Excluded articles included reviews, study protocols, case reports, editorials, abstracts, oral presentations, and studies not comparing MMF to SOC. Studies assessing MMF in primary biliary or sclerosing cholangitis-autoimmune hepatitis overlap syndromes were also excluded, as were studies using MMF as second-line or rescue treatment, or for patients who were intolerant or refractory to SOC. If different populations were included, only data relevant to treatment-naïve subjects were analyzed, provided that they were described separately.

Two reviewers (A. A. and A. A.) extracted relevant data from each study into a structured sheet using Microsoft Excel version 2410. Extracted data included study year, study design, country, number of patients within each study arm, total follow-up period, and MMF dose.

The two reviewers (A. A. and A. A.) assessed the potential bias in the study design and the methods implemented in the included studies. Cochrane tools for bias assessment were used, namely the RoB-2 tool for randomized controlled trials (RCTs) and the ROBINS-I tool for non-randomized studies.

Review Manager (RevMan), version 5.4. the Cochrane Collaboration 2020 software was used for data analysis and plot drawing. Odds ratios (OR) were used to assess differences between interventions. The Mantel‒Haenszel method was used to estimate the weighted average, provided that the number of available studies was small. The random-effects model was used to account for heterogeneity among the included studies. Statistical significance was set at *p* < 0.05, and for the chi-square test of heterogeneity, a p-value < 0.1 was considered significant. The I^2^ statistic was used to assess the heterogeneity between studies, with values below 25% indicating no heterogeneity, 25–50% indicating low heterogeneity, 50–75% indicating moderate heterogeneity, and values above 75% indicating high heterogeneity. A sensitivity analysis was conducted to check whether using a fixed-effects model instead of a random-effects model would alter the results. The review methods were established and agreed upon by the authors before the commencement of the review. The only deviation was the addition of a sensitivity analysis, which was conducted after excluding one study due to a suspected overlap in participant populations.

## Results

**Fig. 1:**
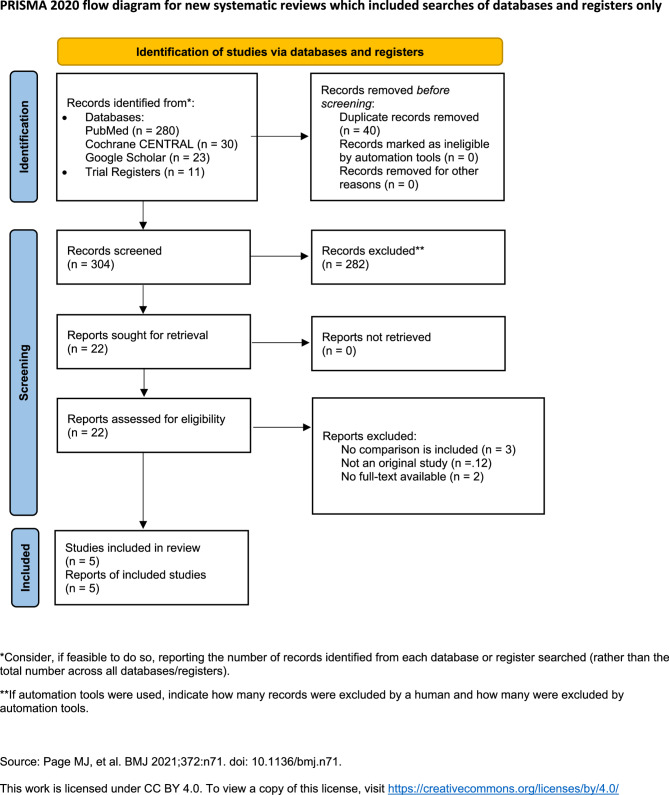
PRISMA flow diagram of the selection process

Till July 2024, the databases’ search yielded 344 studies: 280 from PubMed, 30 from the Cochrane CENTRAL library, 23 from Google Scholar, five from clinicaltrials.gov, and six from WHO-ICTRP. Forty duplicates were removed and the remaining 304 studies were screened for relevance. Of these, twenty-two studies were considered for full-text screening, with five studies, including 613 patients met the inclusion criteria. The PRISMA flow diagram of the search is shown in Fig. 1.

The study by Hlivko et al. [18] was excluded despite the inclusion of 17 treatment-naïve patients receiving a MMF-based regimen; however, pooled results from treatment-naïve patients and those prescribed MMF owing to AZA intolerance were the only reported results. More than 70% of the patients included in our analysis were females, which is consistent with the known demographic prevalence of AIH. Only one of the included studies was an RCT [19]. The four studies included in the quantitative analysis followed the International Autoimmune Hepatitis Group (IAIHG) guidelines [20] for patient diagnosis, excluding those with primary biliary cholangitis/AIH or primary sclerosing cholangitis/AIH variants, burned-out cirrhosis, and other liver diseases, including viral, metabolic, and genetic causes. The characteristics of the included studies are summarized in Table 1. All studies included in this review reported no funding received or disclosed no competing interests.

**Table 1:** Characteristics of the included studies

Author	Year	Country	Design	Treatment duration	Number of participants		Total Number of Patients	Diagnostic Criteria	Female (%)	Age (years)	MMF Dose (mg/day)
					MMF + Pred	AZA + Pred					
Dalekos et al. [21]	2022	Greece	Prospective propensity matching trial	3–5 years	32	32	64	IAIHG	71.90%	54.5	1500–2000
Dalekos et al. [23]	2022	Greece	Prospective cohort study	50 months	183	64	247	IAIHG	73.27%	50	1500–2000
Hlivko et al. [18]	2008	USA	Retrospective cohort study	13 months	16	85	101	AASLD	83%	42.8	500–2000
Snijders et al. [19]	2024	Netherlands + Belgium	RCT	24 weeks	39	31	70	IAIHG	72.90%	57.9	1000–2000
Zachou et al. [15]	2016	Greece	Prospective cohort study	72 months	109	22	131	IAIHG	73.40%	47.5	1500–2000

*Abbreviations*: *AASLD* American association for the study of liver diseases, *AZA* Azathioprine, *IAIHG* International autoimmune hepatitis group, *MMF* Mycophenolate Mofetil, *Pred* Prednisolone, *RCT* randomized controlled trial

All studies compared MMF administered at 1–2 g daily to AZA administered at 1–2 mg/kg/day. Prednisolone was used alongside at an initial dose range of 0.5 to 1 mg/kg daily with similar tapering schedules across all studies. The detailed dosing and tapering schedules for MMF, AZA, and prednisolone in each study are shown in Table 2.

**Table 2:** Treatment regimens of prednisolone, azathioprine, and mycophenolate mofetil across included studies

Study	Prednisolone Starting At Week 1 Dose	Tapering Schedule	AZA Starting Dose	Dose Changes	Final Dose	MMF Starting Dose	Dose Changes	Final Dose
Dalekos et al. [21]	0.5–1 mg/kg/day	Tapered by 5 mg every other week starting from week 3 to week 10 then by 5 mg weekly from week 11 to week 14 then by 2.5 mg weekly thereafter up to complete withdrawal	50 mg started at week 3	Increased by 25 mg every 2 weeks from week 5 to week 8 then by 50 mg on week 9	150 mg	1 g started at week 1	Increased to 1.5 g on week 3 then to 2 g on week 4	2 g
Dalekos et al. [23]	0.5–1 mg/kg/day	Tapered by 5 mg every other week starting from week 3 to week 10 then by 5 mg weekly from week 11 to week 14 then by 2.5 mg weekly thereafter up to complete withdrawal	50 mg started at week 3	Increased by 25 mg every 2 weeks from week 5 to week 8 then by 50 mg on week 9	150 mg	1 g started at week 1	Increased to 1.5 g on week 3 then to 2 g on week 4	2 g
Snijders et al. [19]	40 mg for patients < 80 kg and 60 mg for patients > 80 kg	Tapered down to 15 mg over 5 weeks and then by 2.5 mg weekly over another 5 weeks then the patients were maintained on 5 mg from week 11 to the end of the study	50 mg started at week 5	Increased by 50 mg on week 7	100 mg	1 g started at week 5	Increased to 2 g on week 7	2 g
Zachou et al. [15]	0.5–1 mg/kg/day	5 mg per week till the dose of 15 mg and then the tapering rate was 2.5 mg per week according to the biochemical and clinical response until complete withdrawal	1.5 mg/kg/day	Unclear	2 mg/kg/day	1 g/day	Gradually increased to 1.5–2 g/day after 3 weeks	1.5–2 g daily

*Abbreviations*: *AZA* Azathioprine, *MMF* Mycophenolate Mofetil

### Effect of MMF on complete biochemical remission response (CBR)

**Fig. 2:**
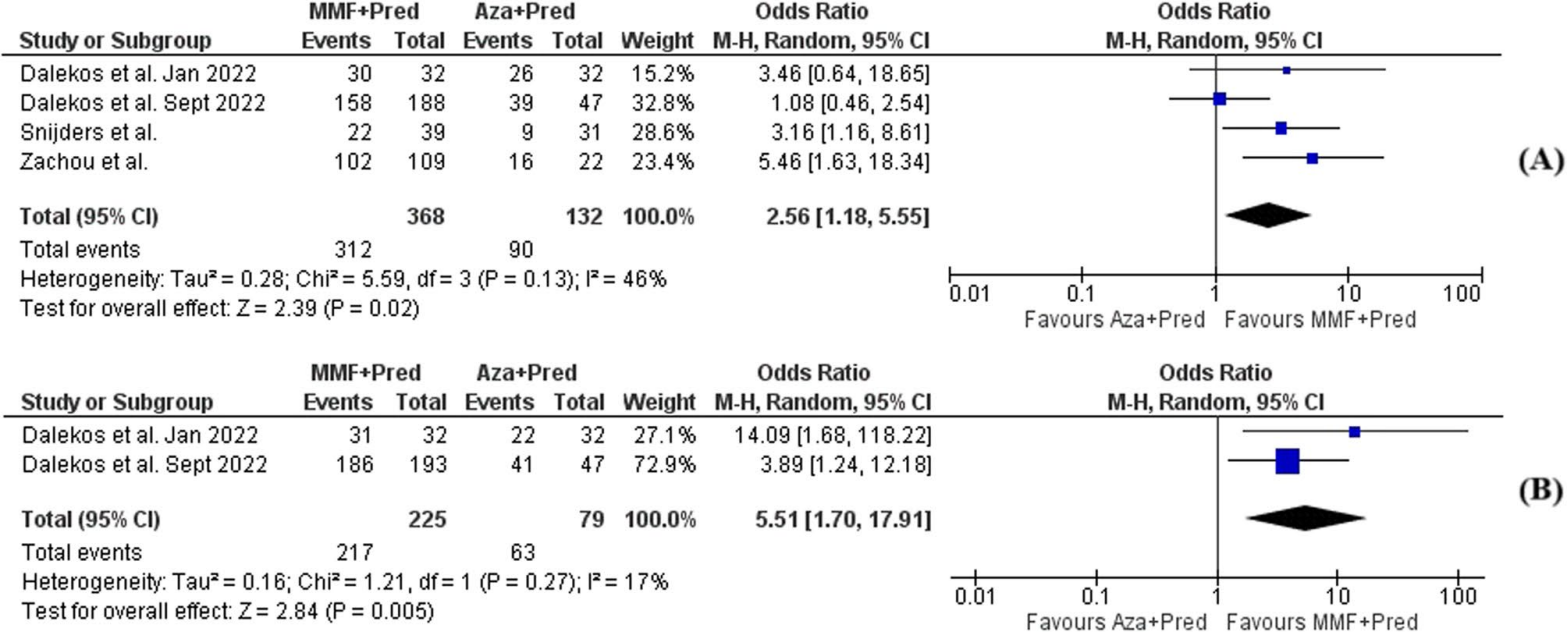
Forest plot of (**A**) short-term and (**B**) long-term complete biochemical remission achieved by mycophenolate mofetil vs. azathioprine both combined with prednisolone in subjects with autoimmune hepatitis

Compared to SOC, MMF combined with prednisolone significantly improved short-term CBR (OR, 2.56; 95% CI, [1.18–5.55], *p* = 0.02, I^2^ = 46%) (Fig. 2), indicating that MMF/prednisolone was associated with a higher rate of achieving short-term CBR.

Only two studies assessed long-term CBR, where significantly higher CBR rates were observed using MMF/prednisolone (OR, 5.51; 95% CI, [1.7–17.91], *p* = 0.005, I^2^ = 17%) (Fig. 2).

### Effect of MMF on non-response rate

**Fig. 3:**
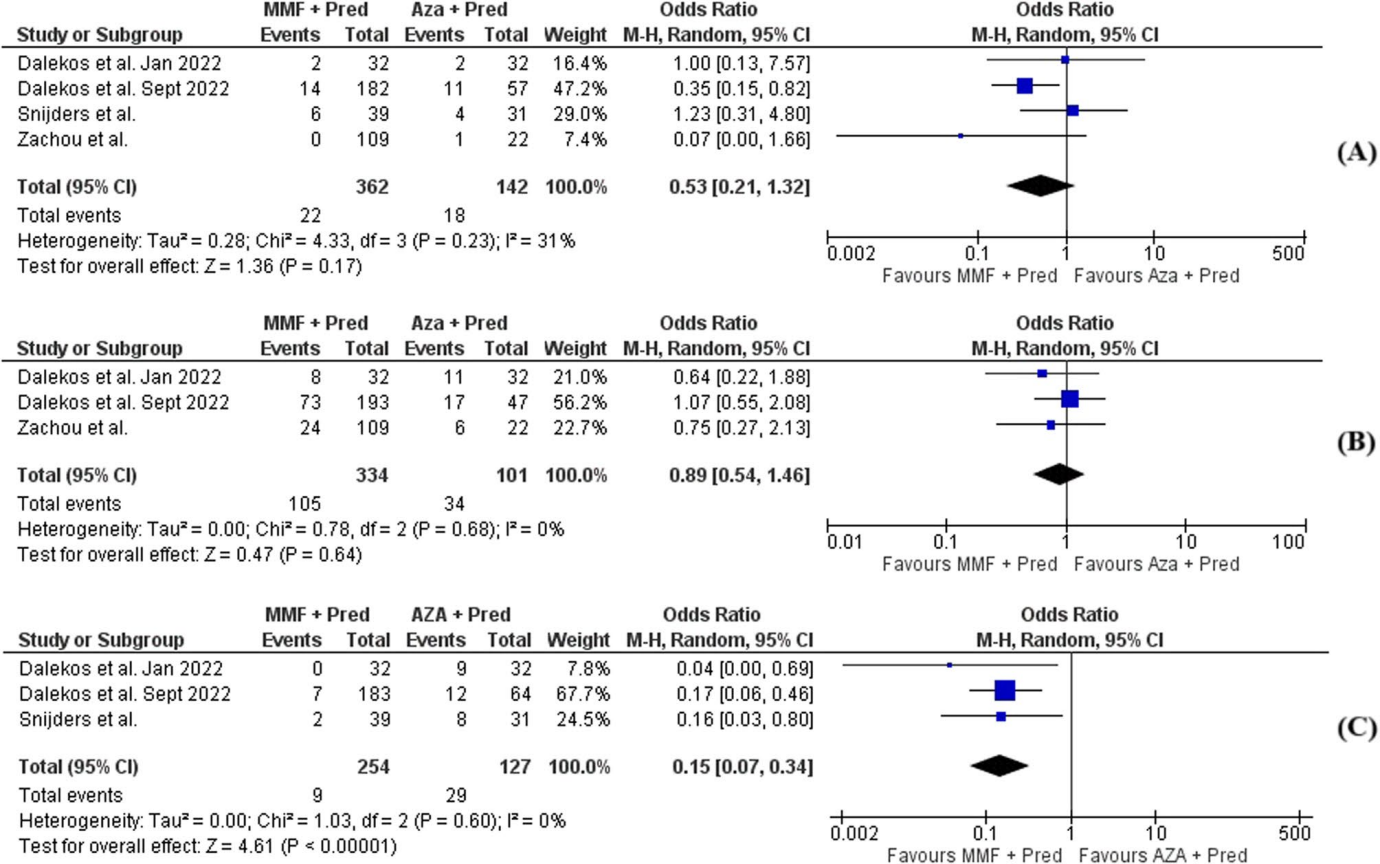
Forest plot of (**A**) nonresponse rate assessed 4 weeks after immunosuppression initiation, (**B**) relapse rate following corticosteroid withdrawal in mycophenolate mofetil vs. azathioprine-treated subjects with AIH and (**C**) rates of treatment discontinuation as a result of serious adverse effects or treatment intolerance

MMF/prednisolone regimens did not significantly differ from SOC (OR, 0.53; 95% CI, [0.21–1.32], *p* = 0.17; I^2^ = 31%) (Fig. 3). It is worth noting that non-response was assessed 4 weeks after initiation, as these drugs were started a few weeks after prednisolone administration in some studies (Table 2).

### Effect of MMF on relapse rate

Relapses following corticosteroid withdrawal in treatment-naïve AIH patients treated with either MMF or AZA showed no significant differences (OR, 0.89; 95% CI, [0.54–1.46], *p* = 0.64; I^2^ = 0%) (Fig. 3).

### Effect of MMF on patient safety

A comparison of the safety of MMF/prednisolone and AZA/prednisolone showed significantly fewer patients requiring treatment discontinuation due to serious adverse effects or intolerance to the MMF regimen (OR, 0.15; 95% CI, [0.07–0.34], *p* < 0.00001, I^2^ = 0%) (Fig. 3). Detailed descriptions of the serious adverse effects leading to treatment discontinuation are presented in Table 3. Adverse events included severe infections, malignancy, rash, peripheral edema, and neutropenia for MMF and severe infections, severe gastrointestinal adverse effects, hepatotoxicity, and myelotoxicity for AZA (Table 3).

**Table 3:** Adverse effects requiring treatment discontinuation in included studies

Study	MMF + Pred group	Total events	AZA + Pred group	Total events
Dalekos et al. [21]	None	0/32	Severe lower respiratory tract infection (*n* = 1) Severe herpes simplex virus stomatitis (*n* = 1) Leukopenia (*n* = 2) Thrombocytopenia (*n* = 1) Marked increase in hepatic transaminases (*n* = 4)	9/32
Dalekos et al. [23]	Intolerance (rash development) (*n* = 1) Severe infections (*n* = 4) Lymphoma (*n* = 1) Melanoma (*n* = 1)	7/183	Intolerance (myelotoxicity and hepatotoxicity) (*n* = 9) Severe infections (*n* = 3)	12/64
Snijders et al. [19]	Severe peripheral oedema (*n* = 1) Neutropenia (*n* = 1)	2/39	Malaise (*n* = 1) Drug-induced liver injury, fever and thrombocytopenia (*n* = 1) Influenza B pneumonia (*n* = 1) Mortality (*n* = 1) Severe gastrointestinal symptoms (nausea and vomiting) (*n* = 4)	8/31
*Abbreviations*: *AZA* Azathioprine, *MMF* Mycophenolate Mofetil, *Pred* Prednisolone				

Regarding the safety of MMF in females of childbearing age, all four included trials consistently reported that MMF is absolutely contraindicated during pregnancy and that women of childbearing potential should use effective contraception throughout the study period. One of the four studies also extended this precaution to male participants who were planning to conceive, advising them to use effective contraception as well [21]. The remaining three trials adopted stricter measures, requiring a negative pregnancy test prior to enrollment and mandating the use of dual contraception from the initiation of treatment until six months after its discontinuation [19, 22, 23].

Notably, the 2016 study classified both MMF and AZA as FDA pregnancy category D medications [21]. In contrast, the two trials published in 2022 highlighted the greater teratogenic potential of MMF compared to AZA [22, 23]. The most recent trial from 2024 emphasized that AZA is considered safe during pregnancy and may be a suitable treatment option for women in this period [19].

### Risk of bias assessment

The RCT by Snijders et al. showed a high overall risk of bias as an open-label trial, as assessed using the RoB-2 criteria. The remaining three studies had an overall moderate risk of bias according to the ROBINS-I criteria. The detailed bias assessment results are shown in Supplementary Table 2. Publication bias could not be assessed using funnel plots or Egger’s test because the number of included studies was small (less than 10).

### Sensitivity analysis

Using a fixed-effects model instead of a random-effects model did not alter the results, except for the non-response rate. The fixed-effects model showed that MMF in combination with prednisolone had significantly lower rates of non-response compared to SOC (OR, 0.51; 95% CI, [0.27–0.99], *p* = 0.04). As three of the four included studies were conducted at the same center in Greece, we suspected a potential overlap in the study population. To address this, we conducted a sensitivity analysis by excluding one of the studies that had a coinciding timeframe. Exclusion of this study did not alter the outcomes of our analyses.

## Discussion

To our knowledge, this is the first meta-analysis to compare the efficacy and safety of a MMF-based regimen versus SOC treatment as first-line treatment for AIH in treatment-naïve patients in terms of CBR, non-response rate, relapse rate, and adverse events requiring treatment discontinuation. In 2019, a meta-analysis encompassing eight studies highlighted the beneficial effects of MMF in managing AIH. However, it included both patients using MMF combined with steroids as first-line therapy and those who switched from AZA-based SOC therapy to MMF in case of AZA intolerance as second-line treatment [5]. In October 2024, the Hellenic Association for the Study of the Liver (HASL) clinical practice guidelines [6] recommended the use of MMF as first-line treatment in combination with steroids and considered it a superior alternative to AZA-based regimens. This recommendation was based on the results of a recently published RCT [19] that confirmed the superior results of MMF in previously published observational studies [15, 18, 22–24]. In this review, data from four studies, including 512 patients followed for 24 weeks to 5 years, were collected and analyzed. The primary findings were higher rates of short- and long-term CBR and a better safety profile with fewer patients requiring treatment discontinuation due to adverse events for MMF-based regimens.

Most patients in our meta-analysis were females, particularly those in the fifth and sixth decades of life. This aligns with the reported incidence and prevalence of AIH among adult females globally [7]. To ensure accuracy and consistency, the study by Hlivko et al. [18] was excluded from the quantitative analysis because it mixed the results from treatment-naïve patients with those who were intolerant to AZA and prescribed MMF. Across the four studies included, patients met the simplified IAIHG diagnostic criteria, showing laboratory abnormalities in liver transaminases, absence of viral hepatitis, elevated serum immunoglobulin G, abnormal serology with increased autoantibody levels, and supportive histological findings [20].

Moreover, our meta-analysis revealed promising outcomes among treatment-naïve patients receiving MMF/prednisolone, showing significantly higher rates of both short-term (≤ 6 months) and long-term (≥ 1 year) CBR with low to no heterogeneity. This finding aligns with that reported by Yu ZJ et al., who highlighted higher remission rates in patients receiving MMF combined with prednisolone as first- or second-line compared to SOC treatment [5].

However, it has been previously reported that MMF has lower efficacy if used as second-line therapy, especially in patients with cirrhosis combined with AIH, due to disease resistance rather than reduced MMF effectiveness [25, 26]. Ngu et al. identified that failure to achieve complete normalization of ALT at 6 months, hypoalbuminemia, and age < 20 or > 60 years at diagnosis are indicators of poorer outcomes and predictors of liver-related mortality or transplantation need [27]. This indicates that achieving a positive CBR will likely lead to better disease prognosis. Dalekos et al. [23] reported that patients treated with MMF showed higher rates of short- and long-term CBR and lower non-response rates, even with concurrent autoimmune diseases and positive antibodies against soluble liver/liver pancreas antigens.

Regarding the non-response rate, MMF treatment had comparable results to SOC using the random-effect model but showed significantly lower non-response rates in the fixed-effects model. This difference may be due to the greater weight imparted by the fixed-effect model in the Dalekos et al. [23] study, which had a large sample size and reported lower non-response rates with MMF-based therapy [28]. Zachou et al. [24] supported these findings, noting that MMF as a first-line therapy for treatment-naïve AIH patients is effective in achieving and maintaining remission with low non-response rates. However, Snijders et al. [17] reported comparable non-response rates between MMF and AZA at 4 and 8 weeks. Moreover, a larger meta-analysis, including 583 participants and comparing SOC treatment with MMF-based regimens, also confirmed lower rates of non-response among those receiving MMF-based regimens [5].

During AIH management, steroid tapering may lead to relapse after the achievement of CBR [29]. According to the IAIHG criteria, relapse is defined as the reappearance of ALT levels more than three times the upper limit of normal after prior CBR; however, milder elevations and/or increased IgG levels may sometimes occur. Comparable results were observed in our meta-analysis for either MMF or AZA combined with steroids with respect to the rate of relapse following corticosteroid withdrawal in treatment-naïve patients with AIH. Zachou et al. [15] reported that neither achieving complete response nor experiencing prior relapse during treatment affected remission maintenance after treatment discontinuation. Only the duration of MMF therapy was the strongest independent predictor, with longer treatment duration correlating with lower relapse rates after steroid tapering [15]. Interestingly, patients treated with MMF were more likely to discontinue steroids compared to those treated with AZA [9, 30]. Current guidelines recommend steroid discontinuation after at least three years of treatment and at least two years of sustained CBR to minimize the risk of relapse [29].

Also, the updated EASL clinical practice guidelines recommend that corticosteroid tapering be guided by biochemical response, particularly transaminase normalization, to avoid under-treatment or relapse due to premature withdrawal [31]. Therefore, an additional consideration is the variability in corticosteroid tapering protocols across included studies. While only one trial [15] explicitly adjusted tapering based on clinical and biochemical response, the rest of the included trials followed fixed schedules or did not clearly specify their tapering criteria [19, 22, 23]. Nonetheless, all studies used comparable treatment targets and applied the same tapering strategy within both treatment arms. Therefore, while these differences may introduce minor clinical heterogeneity, they are unlikely to have significantly influenced the direction or consistency of the pooled treatment effect.

Histological remission represents an important therapeutic objective in AIH and is defined by a modified Histological Activity Index (mHAI) score of < 4/18 that could be evaluated at least 12 months following the initiation of immunosuppressive therapy or during any time point in subsequent follow-up [6]. Despite its clinical relevance, histological outcomes were sparsely and inconsistently reported across the studies included in this meta-analysis. In the study by Dalekos et al. [23], liver biopsy results were available for only 48 out of 247 patients at follow-up, as histological evaluation was restricted to patients who were eligible for treatment withdrawal and consented to the biopsy, highlighting the procedure’s limited applicability due to ethical and acceptability concerns. Additionally, Dalekos et al. propensity-matched trial [22], biopsy data were available for only 4 patients before and after treatment. A third included study by Zachou et al. [15] employed a different histological scoring system, the original Knodell Histologic Activity Index, rather than mHAI, which precluded meaningful comparison between MMF and AZA on histological level.

MMF showed a favorable safety profile, with fewer patients needing treatment discontinuation owing to serious adverse events or intolerance. Consistent with these findings, Zachou et al. reported minimal serious adverse events among MMF-treated patients [24]. Furthermore, Snijders et al. [19] reported higher discontinuation rates among AZA-treated patients due to drug-related adverse events, such as nausea and vomiting [19]. MMF’s favorable safety profile extended to its use as a second-line treatment in another meta-analysis that reported low adverse event rates and, accordingly, low discontinuation rates [32]. Kolev et al. [33] reported that only 8% discontinuation because of adverse events was observed among the included patients. Importantly, reproductive safety must be considered when selecting immunosuppressive therapy. MMF is highly teratogenic, absolutely contraindicated during pregnancy and should be stopped at least 12 weeks prior conception [34]. Its use requires effective contraception in both women of childbearing age and in men planning to father a child. In contrast, AZA is considered safe for use during pregnancy, particularly in cases where the benefits outweigh the potential risks [34]. Recent guidelines recommend the continuation of AZA therapy during pregnancy, as it is associated with better maternal and fetal outcomes. However, given its classification as a category D drug, appropriate counseling regarding potential risks is essential [34, 35]. This important safety difference supports the preferential use of AZA over MMF in patients of reproductive age [19]. Accordingly, in three of the included trials female patients were required to test negative for pregnancy at screening and to use, or agree to use, two effective forms of contraception, such as partner condoms, hormonal methods, diaphragms, copper intrauterine device, sponges, or spermicides [15, 22, 23]. In the study by Snijders et al. [19], instead of mandatory contraceptive measures, females of childbearing potential and men intending to conceive were advised about MMF’s potential teratogenicity and were counseled to maintain effective contraception during the study period.

Our meta-analysis, combined with other real-world studies, propensity-matching trials, and meta-analyses, suggests that MMF is a reliable and effective first-line treatment option apart from AZA for inducing and maintaining remission. It should be emphasized that recommendations for induction treatment with AZA-based regimens were based on RCTs conducted in the past 50 years with the inevitable inherent problems of no investigation for HCV, whereas the criteria of response were largely different from those recently endorsed by the IAIHG [30], with the last report published almost 30 years ago [36–38].

One of the included studies, Dalekos et al. [22] employed a strict propensity score matching approach to reduce selection bias, taking into account multiple clinical, biochemical, and histological factors that could affect the response to treatment. While this strengthens internal validity, as acknowledged by the authors themselves, such a design cannot fully eliminate bias from unmeasured confounders such as treatment adherence, timing of therapy initiation or other undocumented factors. Moreover, unmatched patients in this trial exhibited significant differences in disease duration, fibrosis score, acute presentation and IgG levels. Therefore, although the study contributes valuable real-world data, its observational nature may still influence the pooled treatment effect in this meta-analysis.

This meta-analysis has several limitations: few studies were included, and large-scale RCTs data was lacking. Notably, the three non-randomized trials included in this review were conducted at a single center in Greece, raising concern about potential population overlap; efforts to contact the authors for clarification were unsuccessful. Furthermore, data on histological remission using standardized mHAI criteria were either missing or inconsistently reported across the included studies, thereby limiting the ability to fully evaluate the complete disease remission. Besides, the absence of Child-Turcotte-Pugh and Model for End-Stage Liver Disease (MELD) scores in most included studies limited our ability to assess treatment outcomes in relation to baseline liver disease severity, with only one study reporting significant MELD reduction in MMF and AZA groups but without significant between-group differences [19]. The overall moderate to high bias in the studies included increases the need for RCTs to confirm MMF’s promising results as a first-line treatment in AIH management. Although one study used strict propensity score matching [22], residual confounding from unmeasured factors cannot be ruled out. However, the rarity of the disease and therapeutic bias in recommending AZA-based regimens as a first-line treatment option for AIH enhances the significance of the results of this meta-analysis. Importantly, none of the studies included provided sufficient economic data to evaluate the cost-effectiveness of MMF as a first-line treatment option underscoring the need for future research addressing this important aspect.

## Conclusion

Compared to AZA-based regimens, MMF-based first-line therapy for AIH appears to be a more promising, effective, and safe treatment option, yielding higher CBR rates and fewer serious adverse events requiring treatment discontinuation.

## Supplementary Information

Supplementary Material 1.

Supplementary Material 2.

## Data Availability

The datasets generated during and/or analysed during the current study are available from the corresponding author upon reasonable request.

## Abbreviations

AASLD: American Association for the Study of Liver Diseases
AIH: Autoimmune hepatitis
ALT: Alanine aminotransferase
AST: Aspartate aminotransferase
AZA: Azathioprine
CBR: Complete biochemical remission
CI: Confidence interval
EASL: The European Association for the Study of Liver Disease
HASL: The Hellenic Association for the Study of the Liver
IAIHG: International Autoimmune Hepatitis Group
mHAI: Modified Histological Activity Index
MMF: Mycophenolate Mofetil
OR: Odds ratio
PRISMA: Preferred Reporting Items for Systematic Reviews and Meta-Analysis
SOC: Standard of care
WHO-ICTRP: World Health Organization International Clinical Trials Registry Platform

## References

[1] National Institutes of Diabetes and Digestive and Kidney Diseases (NIDDK). Symptoms & Causes of Autoimmune Hepatitis - NIDDK. Available from: https://www.niddk.nih.gov/health-information/liver-disease/autoimmune-hepatitis/symptoms-causes. Cited 24 Jun 2024.

[2] Linzay CD, Sharma B, Pandit S. StatPearls. Treasure Island (FL): StatPearls Publishing; 2024. Autoimmune Hepatitis. 2023. Available from: https://www.ncbi.nlm.nih.gov/books/NBK459186/. 29083819

[3] European Association for the Study of the Liver. EASL Clinical Practice Guidelines: Autoimmune hepatitis. J Hepatol. 2015;63(4):971–1004. Available from: 10.1016/j.jhep.2015.06.030. 26341719 10.1016/j.jhep.2015.06.030

[4] Mercado LA, Gil-Lopez F, Chirila RM, Harnois DM. Autoimmune Hepatitis: A Diagnostic and Therapeutic Overview. Diagnostics. 2024;14(4). Available from: https://www.mdpi.com/2075-4418/14/4/382. 10.3390/diagnostics14040382 PMC10887775 38396421

[5] Yu ZJ, Zhang LL, Huang TT, Zhu JS, He ZB. Comparison of mycophenolate mofetil with standard treatment for autoimmune hepatitis: a meta-analysis. Eur J Gastroenterol Hepatol. 2019;31(7):873–7. Available from: 10.1097/MEG.0000000000001367. 10.1097/MEG.0000000000001367 31150366

[6] Dalekos GN, Koskinas J, Papatheodoridis GV. Hellenic association for the study of the liver clinical practice guidelines: autoimmune hepatitis. Ann Gastroenterol. 2024;37(X):1–32. Available from: 10.20524/aog.2018.0330. 10.20524/aog.2018.0330 PMC6302199 30598587

[7] Hahn JW, Yang R, Moon S, Chang Y, Lee K, Kim A, et al. Global incidence and prevalence of autoimmune hepatitis, 1970–2022 : a systematic review and meta-analysis. eClinicalMedicine. 2023;65:102280. Available from: 10.1016/j.eclinm.2023.102280. 37876996 10.1016/j.eclinm.2023.102280 PMC10590724

[8] Bittermann T, Lewis JD, Levy C, Goldberg DS. Goldberg DS. Sociodemographic and geographic differences in the US epidemiology of autoimmune hepatitis with and without cirrhosis. Hepatology. 2023;77(2):367–78. Available from: https://journals.lww.com/hep/fulltext/2023/02000/sociodemographic_and_geographic_differences_in_the.8.aspx. 35810446 10.1002/hep.32653 PMC9829924

[9] Mack CL, Adams D, Assis DN, Kerkar N, Manns MP, Mayo MJ, et al. Diagnosis and Management of Autoimmune Hepatitis in Adults and Children: 2019 Practice Guidance and Guidelines From the American Association for the Study of Liver Diseases. Hepatology. 2020;72(2):671–722. Available from: https://journals.lww.com/hep/fulltext/2020/08000/diagnosis_and_management_of_autoimmune_hepatitis.24.aspx. 31863477 10.1002/hep.31065

[10] Gatselis NK, Zachou K, Loza AJM, Cançado ELR, Arinaga-Hino T, Muratori P, et al. Prevalence and significance of antimitochondrial antibodies in autoimmune hepatitis (AIH): Results from a large multicentre study of the International AIH Group. Eur J Intern Med. 2023;116(43):50. Available from: https://www.sciencedirect.com/science/article/pii/S0953620523001978. 10.1016/j.ejim.2023.06.001 37302951

[11] American Liver Foundation. Autoimmune Hepatitis: Symptoms & Treatments. Available from: https://liverfoundation.org/liver-diseases/autoimmune-liver-diseases/autoimmune-hepatitis-aih/. Cited 26 Jun 2024.

[12] National Institutes of Diabetes and Digestive and Kidney Diseases (NIDDK). Definition & Facts for Autoimmune Hepatitis - NIDDK. Available from: https://www.niddk.nih.gov/health-information/liver-disease/autoimmune-hepatitis/definition-facts. Cited 24 Jun 2024.

[13] Liberal R, Krawitt EL, Vierling JM, Manns MP, Mieli-Vergani G, Vergani D. Cutting edge issues in autoimmune hepatitis. J Autoimmun. 2016;75:6–19. Available from: 10.1016/j.jaut.2016.07.005. 10.1016/j.jaut.2016.07.005 27502148

[14] Sebode M, Hartl J, Vergani D, Lohse AW. Autoimmune hepatitis: from current knowledge and clinical practice to future research agenda. Liver Int. 2018;38(1):15–22.Available from: 10.1111/liv.13458. 10.1111/liv.13458 28432836

[15] Zachou K, Gatselis NK, Arvaniti P, Gabeta S, Rigopoulou EI, Koukoulis GK, et al. A real-world study focused on the long-term efficacy of mycophenolate mofetil as first-line treatment of autoimmune hepatitis. Aliment Pharmacol Ther. 2016;43(10):1035–47. Available from: 10.1111/apt.13584. 10.1111/apt.13584 26991238

[16] Page MJ, McKenzie JE, Bossuyt PM, Boutron I, Hoffmann TC, Mulrow CD, et al. The PRISMA 2020 statement: an updated guideline for reporting systematic reviews. BMJ. 2021;372:n71. Available from: 10.1136/bmj.n71. 10.1136/bmj.n71 PMC8005924 33782057

[17] Rethlefsen ML, Kirtley S, Waffenschmidt S, Ayala AP, Moher D, Page MJ, et al. PRISMA-S: an extension to the PRISMA statement for reporting literature searches in systematic reviews. Syst Rev. 2021;10(1):39. Available from: 10.1186/s13643-020-01542-z. 10.1186/s13643-020-01542-z PMC7839230 33499930

[18] Hlivko JT, Shiffman ML, Stravitz RT, Luketic VA, Sanyal AJ, Fuchs M, et al. A single center review of the use of mycophenolate mofetil in the treatment of autoimmune hepatitis. Clin Gastroenterol Hepatol. 2008;6(9):1036–40. Available from: 10.1016/j.cgh.2008.04.006. 10.1016/j.cgh.2008.04.006 18586559

[19] Snijders RJALM, Stoelinga AEC, Gevers TJG, Pape S, Biewenga M, Tushuizen ME, et al. An open-label randomised-controlled trial of azathioprine vs. mycophenolate mofetil for the induction of remission in treatment-naive autoimmune hepatitis. J Hepatol. 2024;80(4):576–85. Available from: 10.1016/j.jhep.2023.11.032. 38101756 10.1016/j.jhep.2023.11.032

[20] Hennes EM, Zeniya M, Czaja AJ, Parés A, Dalekos GN, Krawitt EL, et al. Simplified criteria for the diagnosis of autoimmune hepatitis. Hepatology. 2008;48(1):169–76. Available from: 10.1002/hep.22322. 10.1002/hep.22322 18537184

[21] Zachou K, Gatselis NK, Arvaniti P, Gabeta S, Rigopoulou EI, Koukoulis GK, et al. A real-world study focused on the long‐term efficacy of mycophenolate mofetil as first‐line treatment of autoimmune hepatitis. Aliment Pharmacol Ther. 2016;43(10):1035–47. Available from: 10.1111/apt.13584. 10.1111/apt.13584 26991238

[22] Dalekos GN, Arvaniti P, Gatselis NK, Samakidou A, Gabeta S, Rigopoulou E, et al. First results from a propensity matching trial of mycophenolate mofetil vs. Azathioprine in Treatment-Naive AIH patients. Front Immunol. 2022;11(12):798602. Available from: 10.3389/fimmu.2021.798602. 10.3389/fimmu.2021.798602 PMC8787111 35087524

[23] Dalekos GN, Arvaniti P, Gatselis NK, Gabeta S, Samakidou A, Giannoulis G, et al. Long-term results of mycophenolate mofetil vs. azathioprine use in individuals with autoimmune hepatitis. JHEP Reports. 2022;4(12):100601. Available from: 10.1016/j.jhepr.2022.100601. 10.1016/j.jhepr.2022.100601 PMC9674541 36411768

[24] Zachou K, Gatselis N, Papadamou G, Rigopoulou EI, Dalekos GN. Mycophenolate for the treatment of autoimmune hepatitis: prospective assessment of its efficacy and safety for induction and maintenance of remission in a large cohort of treatment-naïve patients. J Hepatol. 2011;55(3):636–46. Available from: 10.1016/j.jhep.2010.12.032. 10.1016/j.jhep.2010.12.032 21238519

[25] Giannakopoulos G, Verbaan H, Friis-Liby IL, Sangfelt P, Nyhlin N, Almer S. Mycophenolate mofetil treatment in patients with autoimmune hepatitis failing standard therapy with prednisolone and azathioprine. Dig Liver Dis. 2019;51(2):253–7. Available from: 10.1016/j.dld.2018.10.004. 30389427 10.1016/j.dld.2018.10.004

[26] Roberts SK, Lim R, Strasser S, Nicoll A, Gazzola A, Mitchell J, et al. Efficacy and safety of mycophenolate mofetil in patients with autoimmune hepatitis and suboptimal outcomes after standard therapy. Clin Gastroenterol Hepatol. 2018;16(2):268–77. Available from: 10.1016/j.cgh.2017.09.063. 10.1016/j.cgh.2017.09.063 29050991

[27] Ngu JH, Gearry RB, Frampton CM, Stedman CAM. Predictors of poor outcome in patients w Ith autoimmune hepatitis: a population-based study. Hepatology. 2013;57(6):2399–406. Available from: 10.1002/hep.26290. 10.1002/hep.26290 23359353

[28] Dettori JR, Norvell DC, Chapman JR. Fixed-Effect vs Random-Effects Models for Meta-Analysis: 3 Points to Consider. Glob Spine J. 2022;12(7):624–6. Available from: 10.1177/21925682221110527. 10.1177/21925682221110527 PMC9393987 35723546

[29] Ohira H, Takahashi A, Zeniya M, Abe M, Arinaga-Hino T, Joshita S, et al. Clinical practice guidelines for autoimmune hepatitis. Hepatol Res. 2022;52(7):571–85. Available from: 10.1111/hepr.13776. 10.1111/hepr.13776 35533021

[30] Dalekos GN, Koskinas J, Papatheodoridis GV. Hellenic association for the study of the liver clinical practice guidelines: autoimmune hepatitis. Ann Gastroenterol. 2019;32(1):1–23. Available from: 10.20524/aog.2018.0330. 10.20524/aog.2018.0330 PMC6302199 30598587

[31] Dalekos G, Gatselis N, Drenth JP, Heneghan M, Jørgensen M, Lohse AW, et al. EASL Clinical Practice Guidelines on the management of autoimmune hepatitis. J Hepatol. 2025. Available from: 10.1016/j.jhep.2025.03.017. 10.1016/j.jhep.2025.03.017 40348684

[32] Santiago P, Schwartz I, Tamariz L, Levy C. Systematic review with meta-analysis: mycophenolate mofetil as a second-line therapy for autoimmune hepatitis. Aliment Pharmacol Ther. 2019;49(7):830–9. Available from: 10.1111/apt.15157. 10.1111/apt.15157 30761563

[33] Kolev M, Diem S, Diem L, Rodrigues SG, Berzigotti A, Stirnimann G, et al. Mycophenolate mofetil as second line treatment in autoimmune hepatitis - A retrospective single center analysis. J Transl Autoimmun. 2022;5:100172. Available from: 10.1016/j.jtauto.2022.100172. 10.1016/j.jtauto.2022.100172 PMC9702977 36451933

[34] Williamson C, Nana M, Poon L, Kupcinskas L, Painter R, Taliani G, et al. EASL Clinical Practice Guidelines on the management of liver diseases in pregnancy. J Hepatol. 2023;79(3):768–828. Available from: 10.1016/j.jhep.2023.03.006. 10.1016/j.jhep.2023.03.006 37394016

[35] Sarkar M, Brady CW, Fleckenstein J, Forde KA, Khungar V, Molleston JP, et al. Reproductive Health and Liver Disease: Practice Guidance by the American Association for the Study of Liver Diseases. Hepatology. 2021;73(1). Available from: https://journals.lww.com/hep/fulltext/2021/01000/reproductive_health_and_liver_disease__practice.25.aspx. 10.1002/hep.31559 32946672

[36] Kirk AP, Jain S, Pocock S, Thomas HC, Sherlock S. Late results of the Royal free hospital prospective controlled trial of prednisolone therapy in hepatitis B surface antigen negative chronic active hepatitis. Gut. 1980;21(1):78–83. Available from: 10.1136/gut.21.1.78. 10.1136/gut.21.1.78 PMC1419564 6988304

[37] Summerskill WH, Korman MG, Ammon HV, Baggenstoss AH. Prednisone for chronic active liver disease: dose titration, standard dose, and combination with azathioprine compared. Gut. 1975;16(11):876–83. Available from: 10.1136/gut.16.11.876. 10.1136/gut.16.11.876 PMC1413126 1104411

[38] Johnson PJ, McFarlane IG, Williams R. Azathioprine for long-term maintenance of remission in autoimmune hepatitis. N Engl J Med. 1995;333(15):958–63. Available from: 10.1056/NEJM199510123331502. 10.1056/NEJM199510123331502 7666914

