# Supplementary material for: Mycophenolate mofetil versus azathioprine as a first-line treatment for autoimmune hepatitis: a comparative systematic review and meta-analysis
Source: BMC Gastroenterol. 2025 Aug 22;25:613. doi: 10.1186/s12876-025-04206-1 (PMC12372268; doi:10.1186/s12876-025-04206-1)
Supplement: Supplementary file 1 — Supplementary Material 1. [file 12876_2025_4206_MOESM1_ESM.docx]

**Risk of Bias Assessment for the Included Randomized Controlled Trial**

|  | **Random sequence generation (selection bias)** | **Allocation concealment (selection bias)** | **Blinding of participants and researchers (performance bias)** | **Blinding of outcome assessment (detection bias)** | **Incomplete outcome data (attrition bias)** | **Selective reporting (reporting bias)** | **Other bias** |
| --- | --- | --- | --- | --- | --- | --- | --- |
| **Snijders et al. (19)** |  |  |  |  |  |  |  |

| **Bias** | **Authors’ judgment** | **Support for judgment** |
| --- | --- | --- |
| **Random sequence generation (selection bias)** | Low risk | Patients were randomly assigned, with an allocation ratio of 1:1, to receive open-label MMF or azathioprine 4 weeks after baseline. Centralised balanced-block randomisation was computer-generated (facilitated by Castor EDC), with stratification according to the centre and presence or absence of cirrhosis. |
| **Allocation concealment (selection bias)** | Unclear risk | Description of allocation is not included |
| **Blinding of participants and researchers (performance bias)** | High risk | Open label |
| **Blinding of outcome assessment (detection bias)** | Unclear risk | The study protocol described that outcome assessors will be blinded during the analyses. However, the study report did not confirm that this was done while conducting the study. |
| **Incomplete outcome data (attrition bias)** | Low risk | Analyses were performed at the end of the trial in the intention-to-treat (ITT) population (i.e., all randomly assigned patients who had taken at least one dose of study medication). Patients with missing data at a visit were counted as not having had a response or remission at that visit (non-response imputation). The attrition was reported in each group for the clinical characteristics and features of AIH patients. |
| **Selective reporting (reporting bias)** | Low risk | All prespecified outcomes were reported |
| **Other bias** | Unclear risk | There is a slight difference in the group sizes. This is due to use of centralised balanced-block randomisation with stratification based on centre and on cirrhosis, which resulted in an imbalance. This imbalance may have introduced a degree of sampling bias. |

|  | **Pre-intervention domains** | | **At-intervention domain** | **Post-intervention domains** | | | |
| --- | --- | --- | --- | --- | --- | --- | --- |
|  | **Bias due to confounding** | **Bias in selection of participants into the study** | **Bias in classification of interventions** | **Bias due to deviations from intended interventions** | **Bias due to missing data** | **Bias in measurement of the outcome** | **Bias in selection of the reported result** |
| **Dalekos et al. (21)** | **Moderate risk** | **Low risk** | **Low risk** | **Low risk** | **Low risk** | **Low risk** | **Moderate risk** |
| **Dalekos et al. (23)** | **Moderate risk** | **Low risk** | **Low risk** | **Low risk** | **Low risk** | **Low risk** | **Moderate risk** |
| **Zachou et al. (15)** | **Moderate risk** | **Low risk** | **Low risk** | **Low risk** | **Low risk** | **Moderate risk** | **Low risk** |

**Risk of Bias Assessment for the Included Non-randomized Controlled Trial**
