# Supplementary material for: Mycophenolate mofetil versus azathioprine as a first-line treatment for autoimmune hepatitis: a comparative systematic review and meta-analysis
Source: BMC Gastroenterol. 2025 Aug 22;25:613. doi: 10.1186/s12876-025-04206-1 (PMC12372268; doi:10.1186/s12876-025-04206-1)
Supplement: Supplementary file 2 — Supplementary Material 2. [file 12876_2025_4206_MOESM2_ESM.docx]

**SEARCH STRATEGY**

**PubMed**

("mycophenolic acid"[MeSH Terms] OR ("mycophenolic"[All Fields] AND "acid"[All Fields]) OR "mycophenolic acid"[All Fields] OR ("mycophenolate"[All Fields] AND "mofetil"[All Fields]) OR "mycophenolate mofetil"[All Fields] OR ("mycophenolic acid"[MeSH Terms] OR ("mycophenolic"[All Fields] AND "acid"[All Fields]) OR "mycophenolic acid"[All Fields] OR "cellcept"[All Fields] OR ("mycophenolate"[All Fields] AND "mofetil"[All Fields]) OR "mycophenolate mofetil"[All Fields])) AND ("hepatitis, autoimmune"[MeSH Terms] OR ("hepatitis"[All Fields] AND "autoimmune"[All Fields]) OR "autoimmune hepatitis"[All Fields] OR ("autoimmune"[All Fields] AND "hepatitis"[All Fields]))

**Cochrane Central Library**

Autoimmune hepatitis AND (Mycophenolate mofetil OR Cellcept) in Title Abstract Keyword - (Word variations have been searched)

**Google Scholar**

allintitle: Autoimmune hepatitis AND (Mycophenolate mofetil OR Cellcept)

**ClinicalTrials.gov**

autoimmune hepatitis | mycophenolate mofetil \[CellCept\] (Synonyms of conditions or diseases have been searched)

**International Clinical Trials Registry Platform (ICTRP) search portal**

autoimmune; autoimmune || hepatitis; Hepatitides; hepatitis || mycophenolate; mycophenolate || mofetil; mofetil || cellcept; cellcept; Mycophenolate Mofetil; Mycophenolate Mofetil Hydrochloride; Mycophenolate Sodium; Mycophenolic Acid Morpholinoethyl Ester; Myfortic; RS 61443; RS61443; Sodium Mycophenolate
